# Visual Outcome after Vitrectomy with Subretinal tPA Injection to Treat Submacular Hemorrhage Secondary to Age-Related Macular Degeneration or Macroaneurysm

**DOI:** 10.1155/2021/3160963

**Published:** 2021-12-30

**Authors:** Yasmin Ali Said, Evelien Dewilde, Peter Stalmans

**Affiliations:** Department of Ophthalmology, UZ Leuven, Herestraat 49, Leuven 3000, Belgium

## Abstract

**Purpose:**

To determine the efficacy and safety of 23G transconjunctival sutureless vitrectomy, subretinal injection of tissue plasminogen activator using the EVA Surgical System, and pneumatic displacement with air to treat submacular hemorrhages.

**Methods:**

Retrospective analysis of 93 eyes surgically treated for submacular hemorrhage caused by neovascular AMD or retinal macroaneurysms. *Main Outcome Measures*. Postoperative visual acuity and surgical complications.

**Results:**

After surgery, visual acuity improved after 6 weeks but decreased again at the final postoperative visit at 8 months due to progression of the underlying disease. Complications consisted of 2 cases of retinal pigment epithelial tear, 7 vitreous hemorrhages, 4 hyphema, 6 cases of retinal detachment, and 2 subchoroidal hemorrhages during the follow-up period.

**Conclusions:**

This study suggests that a surgical approach with 23G vitrectomy, subretinal tPA injection, and pneumatic displacement using air may be an effective procedure for submacular hemorrhage displacement in patients with AMD and retinal macroaneurysms. However, visual outcome is limited by the underlying macular pathology. Larger multicenter randomized controlled studies are warranted to determine the therapeutic effect of this surgical approach.

## 1. Introduction

Subretinal hemorrhage is a serious complication of age-related macular degeneration (AMD) and retinal macroaneurysms which prohibits continued treatment with anti-Vascular Endothelial Growth Factor (VEGF) injections and leads to irreversible retinal damage. Without treatment, these patients generally have a poor visual prognosis [1–4].

The hemorrhage interferes with the retinal function through three mechanisms: it blocks the metabolic exchange between the retinal pigment epithelium and the outer retina, fibrin retraction causes mechanical stress on the photoreceptors, and iron release from the blood lysis induces direct neurotoxicity [5, 6]. Left untreated, fibrous tissue proliferation, atrophic scar formation, and appearance of a retinal pigment epithelium tear are the most frequent anatomical outcomes. This natural course of submacular hemorrhage incited the search for a safe and effective method to remove the blood beneath the macula to speed up visual recovery and prevent further damage. Mechanical removal of the blood with a forceps is too traumatic and associated with unacceptable complications [4]. Displacement of the subretinal blood can be attempted by injecting a gas bubble with or without tissue Plasmin Activator (tPA) in the vitreous cavity and face-down positioning of the patient [1]. However, the subretinal penetration of tPA is limited, and the positioning of elderly patients is difficult [2]. As such, another approach using vitrectomy and subretinal tPA injection has been proposed [3, 7]. This technique is mostly performed by injection of a mixture of tPA and gas subretinally [8, 9]. However, there are few data on the effect of subretinal injection of tPA combined with air fill of the vitreous cavity.

## 2. Materials and Methods

The study included 93 eyes of 91 patients who suffered from a subretinal hemorrhage secondary to AMD or macroaneurysm. We reviewed records of patients, admitted at the University Hospitals Leuven between 2012 and April 2021, treated with 23G transconjunctival sutureless vitrectomy with subretinal tPA injection using the DORC EVA Surgical System (Zuidland, the Netherlands) and intravitreal air injection to displace the hemorrhage away from the fovea. Patients were treated when the following criteria were met: unilateral submacular hemorrhage, larger than one disc diameter, but smaller than the area between the two vascular arcades, and preserved vision of more than counting fingers in the fellow eye. Combined cataract surgery consisting of phacoemulsification, and intraocular lens implantation was performed in all phakic patients. A posterior vitreous detachment was created if not already present.

Vials containing 2 mg of tPA (Alteplase, Cathflo, Genentech) were stored refrigerated till one hour before use. During the vitrectomy, the complete contact of the vial was diluted in 8 ml of BSS plus, yielding a dilution of 0.25 mg/ml. A 10 ml syringe was filled with this solution and mounted to the viscous fluid injection (VFI) kit (DORC, the Netherlands). The VFI syringed was mounted to an extendible 41-gauge polyimide beveled tip microcannula (DORC, the Netherlands). The viscous fluid injection system was connected to and controlled via the EVA Surgical System using variable levels of system-controlled pneumatic pressure. The tip of the injection needle was introduced transretinally into the subretinal space in the lower part of the blood clot, i.e., close to the inferior vascular arcade. The injected volume of tPA was between 0.5 and 1 ml and induced a local bullous retinal detachment over the macular area extending inferiorly since the needle was introduced into the lower area of the blood clot. At the end of the surgery, a fluid/air exchange was performed to fill the vitreous cavity filled with air. The patient was instructed to keep a prone position after surgery (no face-down positioning). The air bubble floating in the upper part of the vitreous cavity was intended to push the liquefied blood downwards into the lower part of the iatrogenically created subretinal fluid compartment over the macula. In all patients, intravitreal injections of anti-VEGF agents were started or continued after resorption of the air.

The following patient data were recorded: lens status, pre- and postoperative visual acuity, and complications.

We found that determining the amount of blood displacement on retinal photographs was not possible because often the clot is fragmented into several pieces. This frequently leaves some blood remnants around the macula. If the outline of all the blood clots would be measured, the result would show no improvement after surgery, although the patient has significant functional improvement. Hence, we used visual acuity as the primary outcome since this seemed the most reliable parameter and is also most valuable for the patient.

Follow-up visits were performed 1 day, 1 week, and 6 weeks after the surgery. If visual acuity data from more long-term postoperative visits were available in the patient electronical medical record, these were also collected for statistical analysis.

The Snellen visual acuity measurement was converted to logMAR values before performing the statistical measurements. Afterwards, the results were converted back to Snellen measurements for reporting in the manuscript [4]. A change of visual acuity of ≥0.3 logMAR was considered as significant.

Longitudinal mixed models were used to test the evolution of visual acuity through time. Only 381 data were nonmissing on 1023 possible measurements (62.76% missing values). No multiple imputations were possible because multiple imputation requires a maximum of 25% missing values. We, therefore, used a Maximum Likelihood (ML) approach for the linear mixed model [5]. If the residuals of the model are not normally distributed, the bestNormalize R package was used to transform the outcome and report the results of this last linear mixed model. Maximum Likelihood (ML) uses all available data in the study and produces unbiased estimates of the treatment effect and correct *p* values.

## 3. Results

In total, 93 eyes underwent surgery from 91 patients. Five eyes had a retinal microaneurysm that caused the subretinal hemorrhage, the other 88 eyes (from 86 patients) had underlying AMD. Most of the patients with AMD (96%) also had AMD in the fellow eye in a less severe way. About half of the eyes [10] were pseudophakic, and the other 46 phakic eyes underwent combined cataract surgery.

The majority of eyes were operated within two days after the onset of the hemorrhage (60, 2%), 90.3% of eyes within one week, and all 93 eyes within 14 days.

At the initial examination, visual acuity ranged from Snellen 0.3 to light perception (mean Snellen 0.06). After the surgery, the visual acuity improved ranging from light perception to Snellen 0.8 (mean Snellen 0.16) at the 6-week follow-up visit (*p* value <0.001) (Table 1).

The visual acuity tended to decrease with long-term follow-up; the mean final visual acuity was Snellen 0.08 at 8 months. This long-term decrease of visual acuity resulted from continuous active neovascular membranes, fibrosis, or macular atrophy.

There were no intraoperative complications related to the tPA injection recorded. In all cases, successful penetration of the subretinal space was possible with the creation of a submacular bleb. Because of the pressure control of the VFI system, there was no injection of tPA into the subchoroidal space. There were adverse events recorded during the postoperative follow-up (Table 2). Two patients (2.2%) developed a retinal pigment epithelial tear, seven (7.7%) patients had a vitreous hemorrhage, and four had a hyphema (4.4%) that needed a reintervention to reduce secondary eye pressure elevation. Six patients (6.6%) suffered from retinal detachment, with presentation ranging from 3 days to 8 months postoperatively. Two patients 2.2%) suffered from subchoroidal hemorrhage, with decreased visual acuity to hand movements. Ten subjects (11%) had recurrent subretinal hemorrhage in the follow-up period ranging from 2 weeks to 9 months postoperatively. As these new hemorrhages all developed more than one week postoperatively, they probably resulted from the underlying disease rather than the intervention.

## 4. Discussion

Over the past 20 years, a variety of therapeutic approaches have been proposed for the management of submacular hemorrhages. Many of them include the use of tPA for clot lysis.

Some surgeons have tried mechanical removal of the subretinal neovascular membrane and hemorrhage. In the largest series reported, the Subretinal Surgery Trials, the outcome of surgical intervention was not significantly better than the natural history [4]. Moreover, the procedure was rather traumatic to the retina, and retinal pigment epithelium cells were extracted together with the clot. A high complication rate was reported: 30% retinal detachments.

In the early 1990s, tPA was introduced to facilitate surgical removal by clot liquefaction [5, 6]. However, results were disappointing. Alternatively, less-invasive techniques have been described to pneumatically displace a submacular hemorrhage way from the fovea, without actual removal. A simple technique, using an intravitreally injected expansible gas bubble with postoperative face-down positioning, was proposed [1, 11, 12]. The most widespread method, introduced by Heriot, is the use of an intravitreally injected gas bubble combined with an intravitreal injection of tPA as an adjunct to induce liquefaction of the clot to facilitate its displacement [13–24]. This technique required prolonged face-down positioning which is difficult to perform for elderly patients. Furthermore, it is doubtful whether intravitreal tPA can diffuse under the retina. Also, some studies have investigated the combined effect of tPA and gas subretinally injected [8, 9].

Rabbit studies report that tPA, having a molecular weight of 70 kD, is theoretically too large to cross the neural retina into the subretinal space [2]. The most likely explanation for a subretinal fibrinolytic effect after intravitreal injection of tPA is the existence of microlesions allowing diffusion of tPA into the subretinal space, by stretching of the retina from the expanding subretinal hemorrhage. Whether the amount of tPA penetration is enough to cause dissolution of the subretinal clots is questionable [25]. Hillencamp et al. and Zeeburg et al. have demonstrated that better anatomical results were obtained with subretinal tPA compared to intravitreal tPA [7, 26]. Several beneficial effects of vitrectomy on exudative AMD have been described in the literature. Firstly, there seems to be an association between the course of exudative AMD and the presence of vitreomacular traction [27, 28]. As such, vitreoretinal separation at the time of surgery or postoperatively may have beneficial effects. Secondly, the increase in retinal oxygenation after vitrectomy may also have a positive effect on the course of exudative AMD [29, 30]. On the other hand, surgical intervention may induce complications (vitreous hemorrhage and retinal detachment), and vitrectomy can alter the pharmacokinetics of intravitreally injected drugs, hereby reducing their half life and increasing the frequency of intravitreal injections to treat choroidal neovascularisation [31, 32].

Experimental studies, using a rabbit model of submacular hemorrhage, have shown serious damage to the photoreceptors in a matter of days [5]. Thus, it is assumed that the blood needs to be evacuated as soon as possible [5].

It is evident that the most important prognostic factor for long-term visual outcome is the activity of the AMD, which is not affected by displacing the blood. Merely, the surgical technique described in this manuscript mandates a continued anti-VEGF treatment of the disease since the underlying subretinal neovascular membrane is not removed. Moreover, it must be noted that, after vitrectomy, the pharmacokinetics of anti-VEGF injections is altered, which may require a shorter interval of injections [33]. Case series with long-term follow-up, regardless of the technique used (w/o vitrectomy, w/o tPA), mention short-term visual acuity improvement but long-term decline in visual acuity due to recurrent growth of neovascular membranes or scar formation [3, 17, 19, 21, 34–42]. All of our patients received further treatment with anti-VEGF when the CNV was no longer blocked by blood.

The adverse events that occurred in our patients are similar to those found in other indications for vitrectomy surgery [43–47]. However, there is a higher incidence of hemorrhagic complications, which is probably related to the underlying disease (hemorrhage from neovascular choroidal subretinal vessels).

For this study, a pressure-controlled injection system was used that was directly connected to a surgical system (DORC EVA). This allowed the surgeon to hold the 41G injection needle in the subretinal space, while controlling the injection speed of the liquid using the foot pedal. This foot control injection method omits the need for an assistant to manually inject the liquid and prevented the undesired injection of subchoroidal tPA.

In all patients, the vitreous cavity was filled with air at the end of the surgery, followed by supine positioning. Since air resorption progresses quickly, typically at the first postoperative day, two-thirds of the vitreous cavity is filled with air. The buoyancy of the air bubble in the superior part of the eye will push the liquefied blood downwards into the subretinal fluid bleb that was extended inferiorly during the surgery. Hence, the aim of the surgery is to move the submacular blood clot downwards to displace it from its central position. This study is limited by its noncomparative, nonrandomized retrospective design and relatively short-term patient follow-up. The lack of a control group and, therefore, the inability of this study to compare the outcomes of this surgical procedure with natural history are other major limitations of this study.

## 5. Conclusions

This study indicates that a surgical approach with 23G vitrectomy, subretinal tPA injection using the EVA Surgical System, and pneumatic displacement may be an effective procedure for submacular hemorrhage displacement in patients with AMD and retinal macroaneurysms. The procedure induced a temporary improvement of visual acuity. However, the final visual outcome was limited by progression of the underlying macular pathology.

## Figures and Tables

**Table 1:** Evolution of visual acuity after vitrectomy surgery with subretinal injection of tPA and air tamponade.

Time	Mean ± St. dev. (*N*)
0 days	0.06 ± 0.07 (90)
1 day	0.01 ± 0.00 (92)
3 days	0.02 ± 0.03 (17)
1 week	0.06 ± 0.08 (9)
2 weeks	0.11 ± 0.14 (21)
4 weeks	0.07 ± 0.10 (10)
6–8 weeks	0.16 ± 0.20 (57)
3 months	0.16 ± 0.18 (25)
4 months	0.10 ± 0.11 (20)
6 months	0.10 ± 0.13 (18)
8 months	0.08 ± 0.13 (22)

**Table 2:** Adverse events recorded during the postoperative follow-up.

Description	Number of eyes (%)
RPE tear	2 (2.2%)
Vitreous hemorrhage	7 (7.7%)
Hyphema	4 (4.4%)
Retinal detachment	6 (6.6%)
Subchoroidal hemorrhage	2 (2.2%)
Recurrent subretinal hemorrhage	10 (11%)

## Data Availability

The underlying data are patient data stored in the electronical medical record of the UZ Leuven hospital.
